# OxCOVID19 Database, a multimodal data repository for better understanding the global impact of COVID-19

**DOI:** 10.1038/s41598-021-88481-4

**Published:** 2021-04-29

**Authors:** Adam Mahdi, Piotr Błaszczyk, Paweł Dłotko, Dario Salvi, Tak-Shing Chan, John Harvey, Davide Gurnari, Yue Wu, Ahmad Farhat, Niklas Hellmer, Alexander Zarebski, Bernie Hogan, Lionel Tarassenko

**Affiliations:** 1 Department of Engineering Science, Institute of Biomedical Engineering, University of Oxford, Oxford, UK; 2 Faculty of Computer Science, Electronics and Telecommunications, AGH University of Science and Technology, Krakow, Poland; 3 Dioscuri Centre in Topological Data Analysis, Mathematical Institute, Polish Academy of Sciences, Warsaw, Poland; 4 Department of Mathematics, Swansea University, Swansea, UK; 5 School of Arts and Communication (K3), Malmö University, Malmö, Sweden; 6 Department of Mathematics, University of Padova, Padova, Italy; 7 Mathematical Institute, University of Oxford, Oxford, UK; 8 The Alan Turing Institute, London, UK; 9 American University of Sharjah, Sharjah, United Arab Emirates; 10 Department of Zoology, University of Oxford, Oxford, UK; 11 Oxford Internet Institute, University of Oxford, Oxford, UK

**Keywords:** Infectious diseases, Scientific data

## Abstract

Oxford COVID-19 Database (OxCOVID19 Database) is a comprehensive source of information related to the COVID-19 pandemic. This relational database contains time-series data on epidemiology, government responses, mobility, weather and more across time and space for all countries at the national level, and for more than 50 countries at the regional level. It is curated from a variety of (wherever available) official sources. Its purpose is to facilitate the analysis of the spread of SARS-CoV-2 virus and to assess the effects of non-pharmaceutical interventions to reduce the impact of the pandemic. Our database is a freely available, daily updated tool that provides unified and granular information across geographical regions.

**Table:** 

Design type	Data integration objective
Measurement(s)	Coronavirus infectious disease, viral epidemiology
Technology type(s)	Digital curation
Factor types(s)	
Sample characteristic(s)	Homo sapiens

## Introduction

Characterising the impact of the COVID-19 pandemic and understanding the efficacy of policy interventions requires a comprehensive, well-formatted and easily accessible database. The World Health Organisation and the European Centre for Disease Prevention and Control collect daily statistics about cases and deaths from governmental sources. These aggregated databases are widely used in research but they lack granularity and context. In addition, several academic institutions have curated high quality data sets aiming at capturing variables not included in the aforementioned databases: the Coronavirus Resource Center at John Hopkins University^1^, the Real-time Case Tracker^2^, *The Economist*’s Tracker for COVID-19 Excess Deaths^3^ and the Oxford COVID-19 Government Response Tracker^4^. In constrained research contexts related to the pandemic, these databases prove to be immensely useful to researchers and policy-makers seeking to understand both the causes of the spread and the efficacy of public health interventions. Linking such heterogeneous data is vital to understanding the context which gave rise to the observations and to making inferences at a finer spatial resolution. However, the process of linking relevant data across these sources is complex and requires great care.

**Figure 1:**
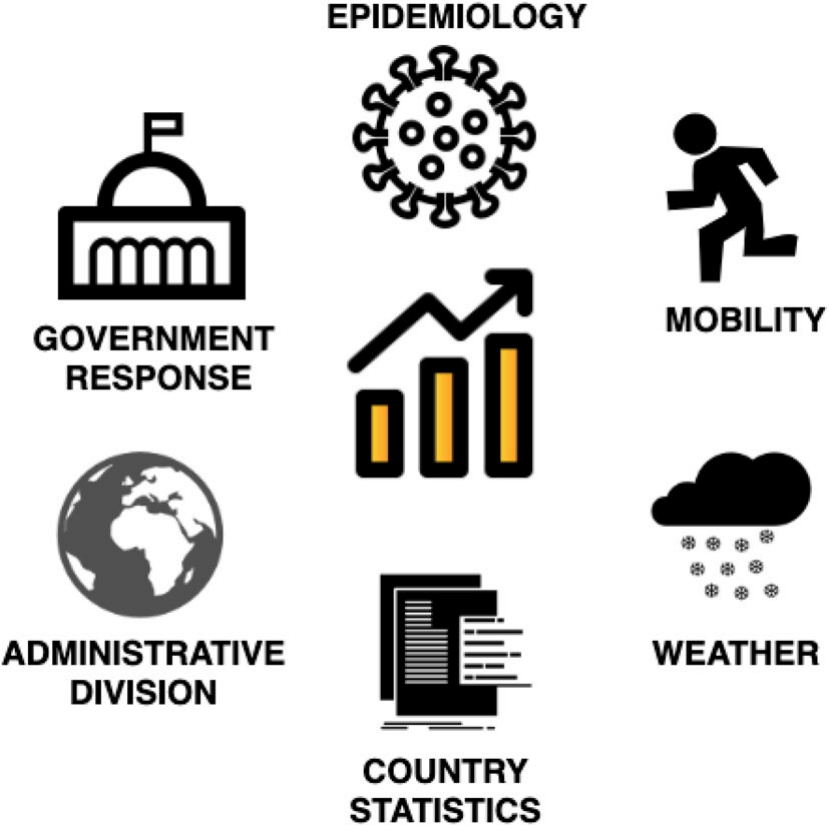
The main types of data categories included in the OxCOVID19 Database.

**Figure 2:**
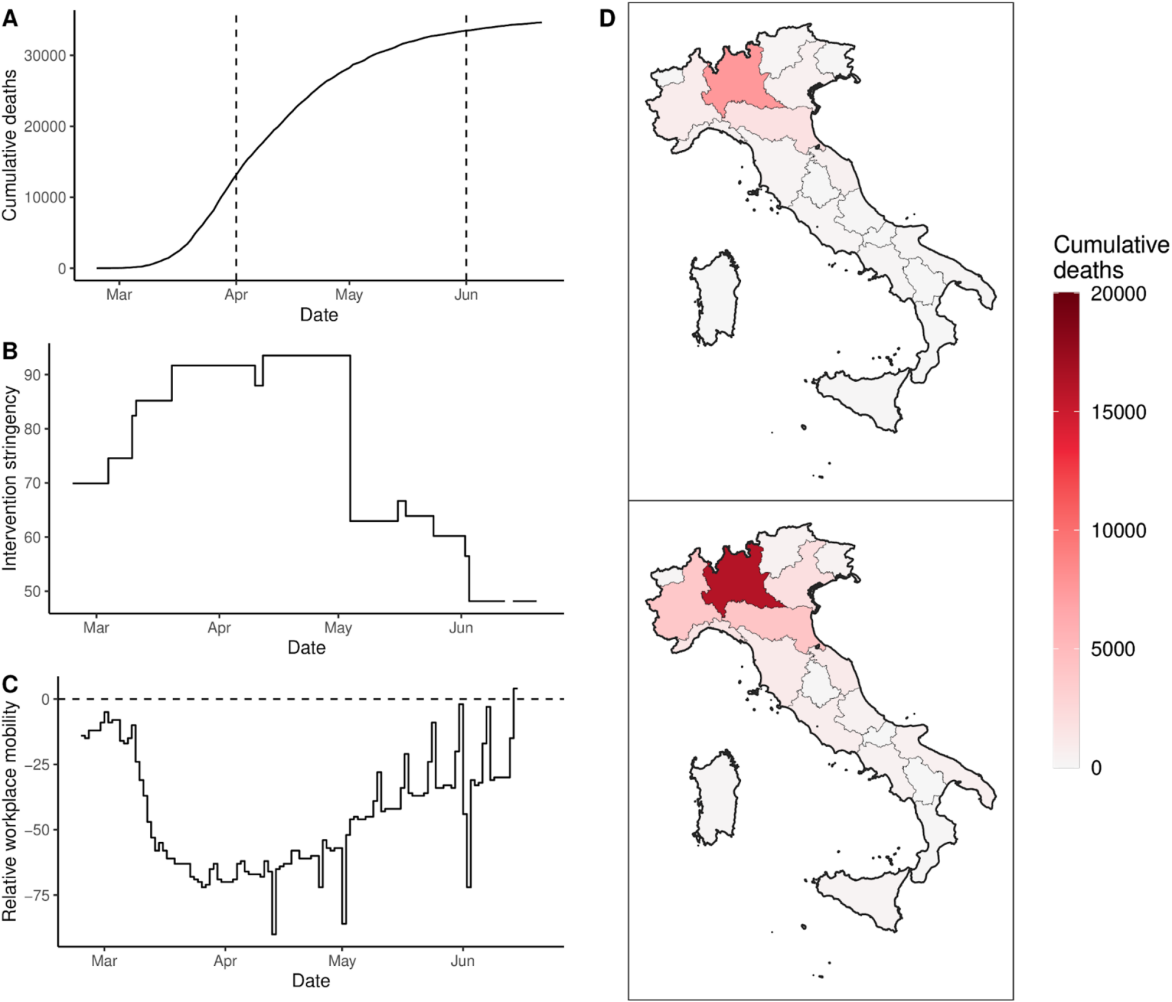
Sample data for Italy demonstrating the data types that are provided by OxCOVID19 Database. (**A**) the cumulative number of deaths through time with two time points (corresponding to 1st of April and 1st of June) indicated by dashed lines. (**B**) the intervention stringency, which is further stratified by the precise type of non-pharmaceutical interventions^4^. (**C**) the relative mobility for workplace activity from Google, the dashed line corresponds to parity with historical values. Panel (**D**) the spatial distribution of the cumulative number of deaths across Italy on the April 1 and June 1, 2020, which corresponds to the dashed lines in (**A**). The choropleth map of Italy was generated with R (https://www.R-project.org, version 4.0.2)^16^. The spatial geometries were obtained using GADMTools^17^ and the figures were generated using ggplot2 (https://ggplot2.tidyverse.org, version 3.3.2)^18^ and ggspatial (https://CRAN.R-project.org/package=ggspatial, version 1.1.3)^19^.

The OxCOVID19 Database aims to link different modalities of data, reported at the national and regional level, including epidemiological information on COVID-19 (confirmed, deaths, recovered, hospitalised, etc.), government response (school closing, economic measures, etc.), mobility (e.g., change in mobility trends of humans in various places), weather (e.g., temperature, humidity, precipitation, etc.), socioeconomic statistics and value surveys (Fig. 1). The database uses an established spatial index GID^5^, which spans several administrative levels. Wherever possible, the OxCOVID19 Database draws upon official government sources, work by university-based or government research groups and data from peer-reviewed scientific papers. The data are provided with the different granular spatial level thereby facilitating a better understanding of how regional characteristics inform the spread of the disease (e.g. Fig. 2). Well-linked and granular data of this type can enable the construction of more accurate models of the pandemic by allowing reliable estimation of the required parameters for relatively small regions, avoiding the process of averaging them on a country level. They also increase our understanding of the efficacy of various interventions at the state and regional levels. Thus, we hope this resource in combination with mathematical modelling and machine learning for data analytics will enhance our understanding of the COVID-19 pandemic and facilitate the development of strategies to reduce the impact on society. Some of the key questions which the OxCOVID19 Database can help to answer include the assessment of the effectiveness of different types of non-pharmaceutical interventions such as government lockdowns, mobility restrictions, and social distancing in reducing the spread of infections^6^.

## Methods

### Data sources

All the data available in the OxCOVID19 Database are collected from publicly available sources including scientific reports^7^, government press releases, briefings, and similar. For the epidemiological data we relied mostly on official government sources including websites and repositories from Ministries of Health, regional Public Health Authorities, university research groups and official social media accounts. Government response data comes from the Coronavirus Response Tracker assembled by researchers from the Blavatnik School of Government at the University of Oxford^4^. For mobility data, we used Community Mobility Reports by Google^8^ and Mobility Trends Reports by Apple^9^. The meteorological data (available from January 1, 2020 onwards) have been made available by the UK Met Office Global Weather Data for COVID-19 Analysis^10^. The socioeconomic statistics and demographics data come from various sources generously made available by the World Values Survey^11^, European Values Study^12^, and the World Bank^13^. A full and updated list of data sources is maintained on https://github.com/covid19db/data.

**Figure 3:**
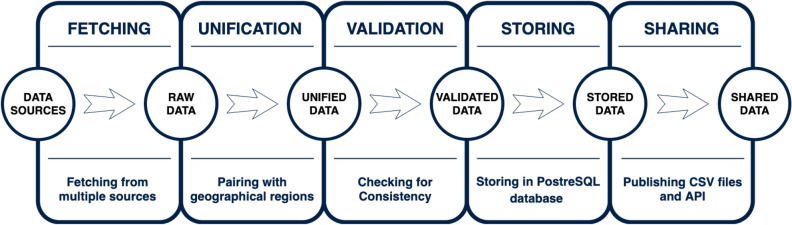
System architecture for OxCOVID19 Database.

There have been numerous challenges in assembling our OxCOVID19 Database. Since this is a “live” database, we had to build a system architecture allowing for daily fetching and validation of the data (see Fig. 3). The datasets used in our work often reported at different levels of geographical division. For example, in the UK, epidemiology was reported to Level 3 while mobility was reported to Level 1 or 2 depending on the source. This presents a substantial challenge when joining the tables. To overcome this problem we introduced a common key, the GID, described in detail in the next section. The sources often change the format, move their location or stop reporting, which present another challenge. To address this, we implemented an automated validation system to detect such issues and alert us when changes need to be made to the fetchers.

Different sources report data at different spatial and temporal resolutions.. We provide information about each source used in our database both on the official project’s GitHub (https://github.com/covid19db/data). At the time of publication we used 53 sources for the EPIDEMIOLOGY table, one for GOVERNMENT_RESPONSE, two for MOBILITY as well as World Values Survey, European Values Study, and the World Bank. As of 22 January 2021, subnational data were being collected at a rate of 1623 EPIDEMIOLOGY records per day, 11617 MOBILITY records per day, and 41319 WEATHER records per day.

### Unifying the data across geographical regions

The OxCOVID19 Database links multimodal data for different levels of administrative division. The largest administrative subdivision of a country will be called the “first-level administrative division”, “first administrative level”, or “Level-1” (e.g., “states” in the USA or “voivodeships” in Poland). The next smaller regions will be described as the “second-level administrative division”, “second administrative level” or “Level-2” (e.g., “counties” in the USA, “powiaty” in Poland); similarly “third-level administrative division”, “third administrative level” or “Level-3” (e.g., “gminy” in Poland). Not every country has a third level (e.g., the USA), some countries do not even have a second level, but we include these where available.

We link data from multiple sources and various levels of administrative subdivision into one relational database using the GID from the Global Administrative Areas (GADM) database as a geographical identifier^5^. The goal of the GADM database is to “map the administrative areas of all countries, at all levels of sub-division”. It is freely available for non-commercial use as is the case here. The GID identifies a geographic area with an alphanumeric string. For example, the string ‘CHN.16.4_1’ can be decoded as follows: the first three letters are the ISO 3166-1 alpha-3 country code for Mainland China; the ‘16’ indicates the Level-1 subdivision, here the province of Jiangxi; the ‘4’ represents the Level-2 division, here Jingdezhen City; finally, the ‘_1’ indicates a version number for the GID, which only changes in the event of major internal reorganisation and allows for backwards compatibility. To each GID a polygon is associated giving the boundaries of the geographical area to an extremely fine resolution. A dedicated table named ADMINISTRATIVE_DIVISIONS contains the GIDs together with their place names and locations, expressed both as a single point and as a polygon. The resolution of the polygon is reduced from that given in GADM in order to conserve space.

Every record, insofar as possible, is matched to a GID or a list of GIDs. This allows the user to match different modalities of data together using GIDs, including across hierarchies. While GID could potentially be used as the main geographical key in our database, we have chosen to introduce some redundancy and use names (as standardised in GADM^5^) of the regions along with their GIDs for ease of use as well as to permit the exceptional absence of GIDs.

Typically, assigning a GID to the region referred to in a record is a straightforward matter. Slight inconsistencies with spelling variations, prefixes, and suffixes can sometimes be an obstacle to carrying out a direct text match, but this requires only limited and obvious manual adjustment. Some records lack the geographical specificity needed to assign a GID, such as where the administrative subdivision is listed as “Unknown”. There are, however, more challenging situations, often relating to administrative reorganisations which have taken place since the release of GADM 3.6. These are best demonstrated by means of example.

#### Handling of boundary changes: some examples

(i) In UK-England, some local authorities (Level-2 units) have undergone boundary changes, with Level-3 units being moved between or promoted to Level-2 units. Each local authority is therefore easily expressed as a list of Level-2 or Level-3 units.
(ii) A similar situation has occurred in Colombia: The Level-1 Department of Cundinamarca (COL.14_1) contains the Capital District, Bogotá, known in GADM as Santafé de Bogotá (COL.14.79_1). However, Bogotá in fact has the status of an independent department. We can still express both Bogotá and Cundinamarca excluding Bogotá by lists of Level-2 GIDs. Unfortunately, Cundinamarca excluding Bogotá contains 114 Level-2 regions. But, we still express it accurately since any alternative would involve a geographical overlap in reporting and possible ambiguity.
(iii) Norway: A reorganisation of counties (Level-1 units) in Norway resulted in several mergers. However, there were also some minor boundary changes. It is possible, as with Colombia, to express all units as lists of Level-2 GIDs. However, it would be extremely cumbersome, and unlike the case of Colombia it is not necessary to avoid geographical overlap, and would result in little gain in accuracy. For simplicity, lists of Level-1 units are used.

#### New organisational schemes: some examples

Where there has been wholesale reorganisation or reporting takes place according to an organisational schema which is not composed of administrative divisions, the situation is less easily handled.

(iv) UK-Scotland: The reporting regions in Scotland are local health boards, which are not compatible with the administrative units, which are local authorities with Level-2 GIDs. We have endeavoured to represent the health boards as accurately as possible with GIDs.
(v) Latvia: The reorganisation of regions in Latvia resulted in much smaller regions which are not included in GADM. The cities and municipalities of Latvia cannot be associated to GIDs.

### Epidemiology

While our goal is to collect epidemiological data on the regional level for as many countries as possible, we initially sought to prioritise countries to include. To determine priority levels we incorporated three criteria: total population, air traffic volume, and number of COVID-19 related deaths. All countries were ordered according to each of the three criteria on 5 May 2020 and the ranks of countries with respect to each criterion are added to give the priority score (i.e., we used a Borda count).

The top 20 countries according to this rank at the time of prioritisation were: United States, China, India, Brazil, United Kingdom, Indonesia, Germany, Turkey, Japan, Spain, Ireland, Russian Federation, France, Italy, Mexico, Pakistan, Belgium, Canada, Iran, Nigeria. We have successfully included regional data for all but Turkey and Iran in the database. At the time of writing, 41 countries have been included at level-1, of which 6 countries are present at level-2, with the United Kingdom at level-3.

### Aggregation of weather variables

The WEATHER table is composed of 47 meteorological variables obtained from the UK Met Office^10^. The variables provide information about temperature, sunshine, humidity and precipitation. This information is sampled daily and reported on a 12 km $\times$ 12 km uniform latitude-longitude grid.

To provide these data in a manner which permits linking with the other tables, we report the mean value for each variable across all grid points contained in Level-1 and Level-2 GADM subdivisions, along with the standard deviation and the number of grid points in the region. We report on a daily basis starting from 1 January 2020.

This level of subdivision was chosen on the basis that almost all Level-2 regions contain a grid point. Where the region contains no grid point, no record is created. This, however, happens in fewer than 0.5% of the cases. Using Level-3 instead of Level-2 would result in a large number of missing records, while using Level-1 would be overly coarse leading to high standard deviations and reduced explanatory power. Further, values for larger geographical units can be obtained by the user by averaging over the smaller subdivisions taking into account the number of points in each region.

#### World Bank data

The World Bank Development Indicators dataset^13^ are an easily accessible set of country-level indicators including economic characteristics (like GDP), quality of healthcare and other metrics. Each record includes an alpha-3 country code (equivalent to GID) allowing them to be linked to our database. Naturally, not all time series are complete for all countries. For ease of use we provide the latest reported values for all indicators. For the full list of available indicators see https://data.worldbank.org/indicator/.

#### Value surveys

We have extracted a number of indicators from the World Values Survey^11^ and European Values Study^12^ including information on the values and beliefs of people; their trust in government, healthcare and scientific institutions; level of poverty; and similar socioeconomic, political, and demographic indicators.

The statistics are aggregated and equipped with the appropriate GID both at the country level and at a regional level where possible. These regions are generally larger than GADM Level-1 and included only for the same 20 countries which were prioritised for epidemiological data.

Our Integrated Values Surveys dataset is obtained by merging together all fully released waves of the World Values Survey and the European Values Study. There is no official release of this integrated dataset—we merged it following the official guidelines^14^ making appropriate adjustments where the guidance has not provided the correct matching.

For each survey question, we report the frequency of each answer. Because each possible answer generates a column, the resulting table has more than 15,000 columns. To reduce the size of the table we instead stored all the statistics for each country/region in a nested dictionary, placed in the column “properties” in the SURVEYS table.

## Data records

The database is available to download at https://covid19.eng.ox.ac.uk/. The data are stored in a PostgreSQL database. CSV extracts from this database are available to access at https://github.com/covid19db/data. A complete archive copy of the database in CSV format as of 31-07-2020 has been stored under the https://doi.org/10.6084/m9.figshare.12746150.

### Common columns for joining tables

The following columns are used to uniquely identify each record and query the database in order to combine different modalities of information: (K1) **source**—an abbreviation indicating the data source; (K2) **date**—ISO 8601 date (YYYY-MM-DD) of the record under consideration; (K3) **GID**; (K4) **country**—English name for a country as it appears in the GADM database, (K5) **countrycode**—ISO 3166-1 alpha-3 country codes, (K6) **adm_area_1**—specifying first-level administrative country subdivision, (K7) **adm_area_2**—specifying second-level administrative country subdivision, (K8) **adm_area_3**—specifying third-level administrative country subdivision. Note that although (K1)–(K3) uniquely identifies each record the additional columns such as country, countrycode or different levels of administrative division permit more user friendly means to query groups or aggregates of the data as necessary. (K6)–(K8) are strings in the Latin alphabet given as they appear in the GADM database unless the region is not associated with a single GID. In most such cases, it is reported as in the original source. In the case of an Upper Tier Local Authority of England, such as the Boroughs of Greater London, for the sake of ease of use, divisions are listed with their names under **adm_area_2**, with **adm_area_3** being NULL.

### Administrative divisions

The ADMINISTRATIVE_DIVISIONS table (see Table 1) contains the geographic features and information associated with each GID, extracted from GADM^5^. It includes six linking columns (K3)–(K8), followed by **countrycode_alpha2**, the ISO 3166-1 alpha-2 country code, **adm_level**, specifying which level of division it is, **adm_area_1_code**, **adm_area_2_code** and **adm_area_3_code**, providing the GID for each higher level administrative division, **properties**, which includes alternative names and identification codes and three geometric features: **latitude** and **longitude**, specifying the centroid of the region, and **geometry**, specifying the simplified boundaries of the region (shapefiles) for mapping purposes.

### Epidemiological data

The EPIDEMIOLOGY table includes all eight linking columns, (K1)–(K8), followed by **tested**—number of tests; **confirmed**—number of confirmed cases; **dead**—number of deaths; **recovered**—number of individuals recovered; **hospitalised**—number of individuals hospitalised; **hospitalised_icu**—number of individuals in Intensive Care Units; **quarantined**—number of individuals quarantined (see Table 2).

### Government response data

The GOVERNMENT_RESPONSE table includes all eight linking columns, (K1)–(K8), followed by a number of indicators (see Table 3), prepared and curated by researchers from the Blavatnik School of Government, University of Oxford^4^. These indicators are grouped into the following categories: containment and closure, economic response and health systems, and miscellaneous policy announcements that do not fit anywhere else.

### Mobility data

The MOBILITY table (see Table 4) includes all eight linking columns, (K1)–(K8), followed by a number of indicators of human mobility as reported by Google^8^. These data are derived from aggregated movements of Android phone users and are stratified by the location of the user: place of work, outdoor parks, recreation areas, grocery markets etc. This table also contains the change in traffic volume reported by Apple^9^ from aggregated tracking of iPhone users of people walking, driving or taking public transit in their communities.

Google measures mobility on any day relative to the median value for each of the five days falling on the same day of the week in the period January 3–February 6, 2020, while Apple measures all data relative to January 13, 2020. The data only describe mobility within particular locations for particular activities. They do not indicate the amount of travel between regions nor do they contain individual-level data.

### Weather data

The WEATHER table (see Table 5) includes all eight linking columns (K1)–(K8) followed by 47 variables including temperature, sunshine, precipitation, air temperature, wind speed etc.

### World Bank

The WORLD_BANK table (see Table 7) includes seven of the eight linking columns (K1), (K3)–(K8) followed by the **indicator_name**, **indicator_code**, **value** and **year**. Each indicator name and the corresponding code relate to one of the 1431 features listed at https://data.worldbank.org/indicator/. The original source provides a series of values from 1960 to 2019. However, here we report only the most recent available value with its year.

### Surveys

The SURVEYS table (see Table 6) includes seven of the eight linking columns (K1), (K3)–(K8) followed by **samplesize** indicating the number of people taking part in the survey for the region under consideration, **properties**, which is a dictionary containing the region/country statistics and **wave**, specifying the particular survey being reported.

## Technical validation

The code used to build the OxCOVID19 Database was developed collaboratively. Working across several GitHub repositories (https://github.com/covid19db) allowed us to share documentation and keep code organised and up to date. We encourage the research community to report any issues they find. Figure 3 shows the system architecture that is being used to collect, unify, store and share the data. We operate more than 70 fetchers to periodically obtain raw data from our sources. This automated process ensures that we collect the most recent data and reduces potential error due to manual entry. The “Unification” step ensures that the names in different tables in the OxCOVID19 Database are consistent across geographical regions. In the “Validation” step a check for consistency is performed. During the storing step, the last timestamp in input data is compared with the current time and if the inserted data are older than 14 days relevant warnings are generated as that may indicate the change in format of the fetched data or some other problem that needs to be fixed. The fetching process is triggered twice a day at 02:00 and 14:00 BST. The sharing process, namely publishing existing data sources to CSV files hosted on GitHub, is triggered four times a day.

## Usage notes

### Data access

We provide several different means of accessing the OxCOVID19 Database. The latest version can be downloaded in CSV format from https://github.com/covid19db/data or https://covid19.eng.ox.ac.uk/ and the archived static version can be accessed from FigShare https://doi.org/10.6084/m9.figshare.12746150. Direct connection to the PostgreSQL database can also be granted upon request.

### Example usage

The basic examples in Python and R showing how to load the data and perform simple analysis are available at https://github.com/covid19db/examples. We would like to acknowledge that the R package for accessing our database, available on CRAN [https://cran.r-project.org/package=oxcovid19], has been developed by the members of the CoMo Consortium^15^.

### Citation advice

The OxCOVID19 Database is the results of many hours of volunteer efforts and generous contributions from many organisations listed in the Methods section under “Data Sources”. We encourage the users of OxCOVID19 Database to cite, along with this article, the underlying sources.

**Table 1:** Schema for ADMINISTRATIVE_DIVISIONS table.

Name	Type	Description
Gid	Array	Unique geographical ID, for more details see gadm.org
Country	Varchar	English name for the country
Countrycode	Varchar	ISO 3166-1 alpha-3 country codes
Countrycode_alpha2	Varchar	ISO 3166-1 alpha-2 country codes
Adm_area_1	Varchar	Level-1 administrative country subdivision
Adm_area_1_code	Varchar	First-level administrative country code subdivision
Adm_area_2	Varchar	Level-2 administrative country subdivision
Adm_area_2_code	Varchar	Second-level administrative country code subdivision
Adm_area_3	Varchar	Level-3 administrative country subdivision
Adm_area_3_code	Varchar	Third-level administrative country code subdivision
Adm_level	Integer	0—for countries level, 1—for regions etc.
Latitude	Float	Geographic coordinate of region’s centroid
Longtitude	Float	Geographic coordinate of region’s centroid
Properties	json	Additional attributes describing region
Geometry	Geometry	Polygon describing geographical area

**Table 2:** Schema for EPIDEMIOLOGY table.

Name	Type	Description
Source	Varchar	Specify data source
Date	Date	Day of the statistics
Gid	Array	Unique geographical ID, for more details see gadm.org
Country	Varchar	English name for the country
Countrycode	Varchar	ISO 3166-1 alpha-3 country codes
Adm_area_1	Varchar	Level-1 administrative country subdivision
Adm_area_2	Varchar	Level-2 administrative country subdivision
Adm_area_3	Varchar	Level-3 administrative country subdivision
Tested	Int	Number of people tested
Confirmed	Int	Number of confirmed cases
Dead	Int	Number of deaths
Recovered	Int	Number of confirmed who recovered
Hospitalised	Int	Number of confirmed who are/have been hospitalised
Hospitalised_icu	Int	Number of confirmed who are/have been in the intensive care
Quarantined	Int	Number of confirmed with home quarantine

**Table 3:** Schema for GOVERNMENT_RESPONSE table.

Name	Type	Description
Source	Varchar	Specify data source
Date	Date	Day of the statistics
Gid	Array	Unique geographical ID, for more details see gadm.org
Country	Varchar	English name for the country
Countrycode	Varchar	ISO 3166-1 alpha-3 country codes
Adm_area_1	Varchar	Level-1 administrative country subdivision
Adm_area_2	Varchar	Level-2 administrative country subdivision
Adm_area_3	Varchar	Level-3 administrative country subdivision
c1_school_closing	Integer	Record closings of schools and universities
c1_flag	Integer	Binary flag for geographic scope
c2_workplace_closing	Integer	Record closings of workplaces
c2_flag	Integer	Binary flag for geographic scope
c3_cancel_public_events	Integer	Record cancelling public events
c3_flag	Integer	Binary flag for geographic scope
c4_restrictions_on_gatherings	Integer	Record limits on private gatherings
c4_flag	Integer	Binary flag for geographic scope
c5_close_public_transport	Integer	Record closing of public transport
c5_flag	Integer	Binary flag for geographic scope
c6_stay_at_home_requirements	Integer	Record orders to “shelter-in-place” and otherwise confine to the home
c6_flag	Integer	Binary flag for geographic scope
c7_restrictions_on_internal_movement	Integer	Record restrictions on internal movement between cities/regions
c7_flag	Integer	Binary flag for geographic scope
c8_international_travel_controls	Integer	Record restrictions on international travel. Note: this records policy for foreign travellers, not citizens
e1_income_support	Integer	Record if the government is providing direct cash payments to people who lose their jobs or cannot work. Note: only includes payments to firms if explicitly linked to payroll/salaries
e1_flag	integer	Binary flag for geographic scope
e2_debtcontract_relief	Integer	Record if the government is freezing financial obligations for households (eg stopping loan repayments, preventing services like water from stopping, or banning evictions)
e3_fiscal_measures	Float	Announced economic stimulus spending. Note: only record amount additional to previously announced spending
e4_international_support	Float	Announced offers of Covid-19 related aid spending to other countries. Note: only record amount additional to previously announced spending
h1_public_information_campaigns	Integer	Record presence of public info campaigns
h1_flag	Integer	Binary flag for geographic scope
h2_testing_policy	Integer	Record government policy on who has access to testing. Note: this records policies about testing for current infection (PCR tests) not testing for immunity (antibody test)
Continued		
h3_contact_tracing	Integer	Record government policy on contact tracing after a positive diagnosis. Note: we are looking for policies that would identify all people potentially exposed to Covid-19; voluntary bluetooth apps are unlikely to achieve this
h4_emergency_investment_in_healthcare	Float	Announced short term spending on healthcare system, e.g. hospitals, masks, etc. Note: only record amount additional to previously announced spending
h5_investment_in_vaccines	Float	Announced public spending on Covid-19 vaccine development. Note: only record amount additional to previously announced spending
m1_wildcard	Varchar	Record policy announcements that do not fit anywhere else
Stringency_index	Float	Calculated as a function of the individual indicators.
Stringency_indexfordisplay	Float	Calculated as a function of the individual indicators.
Stringency_legacy_index	Float	Calculated as a function of the individual indicators.
Stringency_legacy_indexfordisplay	Float	Calculated as a function of the individual indicators.
Government_response_index	Float	Calculated as a function of the individual indicators.
Government_response_index_for_display	Float	Calculated as a function of the individual component indicators.
Containment_health_index	Float	Calculated as a function of the individual indicators.
Containment_health_index_for_display	Float	Calculated as a function of the individual indicators.
Economic_support_index	Float	Calculated as a function of the individual indicators.
Economic_support_index_for_display	Float	Calculated as a function of the individual indicators.
Actions	jsonb	Raw response from Covid Tracker API containing all above indicators with full description stored in JSON format.

**Table 4:** Schema for MOBILITY table.

Name	Type	Short description
Source	Varchar	Specify data source
Date	Date	Day of the statistics
Gid	Array	Unique geographical ID, for more details see gadm.org
Country	Varchar	English name for the country
Countrycode	Varchar	ISO 3166-1 alpha-3 country codes
Adm_area_1	Varchar	Level-1 administrative country subdivision
Adm_area_2	Varchar	Level-2 administrative country subdivision
Adm_area_3	Varchar	Level-3 administrative country subdivision
Transit_stations	Float	Mobility trends reported by Google for transit stations
Residential	Float	Mobility trends reported by Google for places of residence
Workspace	Float	Mobility trends reported by Google for places of work
Parks	Float	Mobility trends reported by Google for places like parks, national parks, public beaches, marinas, dog parks, plazas and public gardens
Retail_recreation	Float	Mobility trends reported Google for places like restaurants, cafes, shopping centers, theme parks, museums, libraries, and movie theaters
Grocery_pharmacy	Float	Mobility trends reported by Google for places like grocery markets, food warehouses, farmers markets, specialty food shops, drug stores, and pharmacies
Transit	Float	The change in volume reported by Apple of people taking public transit in their communities
Walking	Float	The change in volume reported by Apple of people walking in their communities
Driving	Float	The change in volume reported by Apple of people driving taking public transit in their communities

**Table 5:** Schema for WEATHER table.

Name	Type	Description
Source	Varchar	Specify data source
Date	Date	Day of the statistics
Gid	Array	Unique geographical ID, for more details see gadm.org
Country	Varchar	English name for the country
Countrycode	Varchar	ISO 3166-1 alpha-3 country codes
Adm_area_1	Varchar	Level-1 administrative country subdivision
Adm_area_2	Varchar	Level-2 administrative country subdivision
Adm_area_3	Varchar	Level-3 administrative country subdivision
Samplesize	Int	Number of grid points
Precipitation_max_avg	Float	Average of the daily maximum precipitation
Precipitation_max_std	Float	Standard deviation of the daily maximum precipitation
Precipitation_mean_avg	Float	Average of the daily mean precipitation
Precipitation_mean_std	Float	Standard deviation of the daily mean precipitation
Humidity_max_avg	Float	Average of the daily maximum specific humidity
Humidity_max_std	Float	Standard deviation of the daily maximum specific humidity
Humidity_mean_avg	Float	Average of the daily mean specific humidity
Humidity_mean_std	Float	Standard deviation of the daily mean specific humidity
Humidity_min_avg	Float	Average of the daily minimum specific humidity
Humidity_min_std	Float	Standard deviation of the daily minimum specific humidity
Sunshine_max_avg	Float	Average of the daily maximum short wave radiation
Sunshine_max_std	Float	Standard deviation of the daily maximum short wave radiation
Sunshine_mean_avg	Float	Average of the daily minimum short wave radiation
Sunshine_mean_std	Float	Standard deviation of the daily minimum short wave radiation
Temperature_max_avg	Float	Average of the daily maximum temperature
Temperature_max_std	Float	Standard deviation of the daily maximum temperature
Temperature_mean_avg	Float	Average of the daily mean temperature
Temperature_mean_std	Float	Standard deviation of the daily mean temperature
Temperature_min_avg	Float	Average of the daily minimum temperature
Temperature_min_std	Float	Standard deviation of the daily minimum temperature
Windgust_max_avg	Float	Average of the daily maximum wind gust
Windgust_max_std	Float	Standard deviation of the daily maximum wind gust
Windgust_mean_avg	Float	Average of the daily mean wind gust
Windgust_mean_std	Float	Standard deviation of the daily mean wind gust
Windgust_min_avg	Float	Average of the daily minimum wind gust
Windgust_min_std	Float	Standard deviation of the daily minimum wind gust
Windspeed_max_avg	Float	Average of the daily maximum wind speed
Windspeed_max_std	Float	Standard deviation of the daily maximum wind speed
Windspeed_mean_avg	Float	Average of the daily mean wind speed
Windspeed_mean_std	Float	Standard deviation of the daily mean wind speed
Windspeed_min_avg	Float	Average of the daily minimum wind speed
Windspeed_min_std	Float	Standard deviation of the daily minimum wind speed
Cloudaltitude_max_valid	Float	Percentage of points with a valid value of cloudaltitude_max
Cloudaltitude_max_avg	Float	Average of the daily maximum cloud base altitude
Cloudaltitude_max_std	Float	Standard deviation of the daily maximum cloud base altitude
Cloudaltitude_min_valid	Float	Percentage of points with a valid value of cloudaltitude_min
Cloudaltitude_min_avg	Float	Average of the daily minimum cloud base altitude
Cloudaltitude_min_std	Float	Standard deviation of the daily minimum cloud base altitude
Cloudaltitude_mean_valid	Float	Percentage of points with a valid value of cloudaltitude_mean
Cloudaltitude_mean_avg	Float	Average of the daily mean cloud base altitude
Cloudaltitude_mean_std	Float	Standard deviation of the daily mean cloud base altitude
Cloudfrac_max_avg	Float	Average of the daily maximum cloud area fraction
Cloudfrac_max_std	Float	Standard deviation of the daily maximum cloud area fraction
Cloudfrac_min_avg	Float	Average of the daily minimum cloud area fraction
Cloudfrac_min_std	Float	Standard deviation of the daily minimum cloud area fraction
Cloudfrac_mean_avg	Float	Average of the daily mean cloud area fraction
Cloudfrac_mean_std	Float	Standard deviation of the daily mean cloud area fraction

**Table 6:** Schema for SURVEYS table.

Name	Type	Description
Source	Varchar	Data source of the survey
Wave	Varchar	Wave period of the survey
Gid	Array	Unique geographical ID, for more details see gadm.org
Country	Varchar	English name for the country
Countrycode	Varchar	ISO 3166-1 alpha-3 country codes
Adm_area_1	Varchar	Level-1 administrative country subdivision
Adm_area_2	Varchar	Level-2 administrative country subdivision
Adm_area_3	Varchar	Level-3 administrative country subdivision
Samplesize	Int	Number of questions
Properties	Dict	Dictionary containing the region/country statistics.

**Table 7:** Schema for WORLD_BANK table.

Name	Type	Description
Source	Varchar	Specify data source
Gid	Array	Unique geographical ID, for more details see gadm.org
Country	Varchar	English name for the country
Countrycode	Varchar	ISO 3166-1 alpha-3 country codes
Adm_area_1	Varchar	Level-1 administrative country subdivision
Adm_area_2	Varchar	Level-2 administrative country subdivision
Adm_area_3	Varchar	Level-3 administrative country subdivision
Indicator_name	Varchar	Description of the indicator
Indicator_code	Varcar	World Bank indicator code
Value	Float	Most recent non-empty value
Year	Int	Year of the most recent value

## Data Availability

The code for data acquisition and cleaning used in the processing of assembling the OxCOVID19 Database is on the GitHub repository: https://github.com/covid19db.
